# Rhythm Control Better Prevents Dementia than Rate Control Strategies in Patients with Atrial Fibrillation—A Nationwide Cohort Study

**DOI:** 10.3390/jpm12040572

**Published:** 2022-04-03

**Authors:** Jiunn-Cherng Lin, Cheng-Hung Li, Yun-Yu Chen, Chi-Jen Weng, Yu-Shan Chien, Shang-Ju Wu, Chu-Pin Lo, Hui-Chun Tien, Ching-Heng Lin, Jin-Long Huang, Yenn-Jiang Lin, Yu-Cheng Hsieh, Shih-Ann Chen

**Affiliations:** 1 Cardiovascular Center, Taichung Veterans General Hospital, Taichung 40705, Taiwan; horrisong@gmail.com (J.-C.L.); lichcil@yahoo.com.tw (C.-H.L.); scott91695@gmail.com (C.-J.W.); ruby_chien_2@yahoo.com (Y.-S.C.); alanwu0206@gmail.com (S.-J.W.); golden@vghtc.gov.tw (J.-L.H.); epsachen@ms41.hinet.net (S.-A.C.); 2 Department of Internal Medicine, Faculty of Medicine, Institute of Clinical Medicine, National Yang Ming Chiao Tung University, Hsinchu 11217, Taiwan; linyennjiang@gmail.com; 3 Department of Internal Medicine, Taichung Veterans General Hospital Chiayi Branch, Chiayi 60090, Taiwan; 4 Department of Data Science and Big Data Analytics, Providence University, Taichung 43301, Taiwan; cplo@kimo.com; 5 Department of Financial Engineering, Providence University, Taichung 43301, Taiwan; hctien@pu.edu.tw; 6 Heart Rhythm Center, Division of Cardiology, Department of Medicine, Taipei Veterans General Hospital, Taipei 11217, Taiwan; r01847021@gmail.com; 7 Institute of Epidemiology and Preventive Medicine, College of Public Health, National Taiwan University, Taipei 10617, Taiwan; 8 Department of Medical Research, Taichung Veterans General Hospital, Taichung 40705, Taiwan; epid@vghtc.gov.tw; 9 Department of Industrial Engineering and Enterprise Information, Tunghai University, Taichung 40704, Taiwan; 10 School of Medicine, National Chung Hsing University, Taichung 40227, Taiwan

**Keywords:** atrial fibrillation, rhythm control, rate control, stroke, dementia

## Abstract

Background: Atrial fibrillation (AF) increases the risk of dementia. Whether the pharmacological rhythm control of AF can reduce the risk of dementia compared to the rate control strategy remains unclear. We hypothesize that the rhythm control strategy is better than the rate control strategy in preventing dementia. Methods: AF patients aged ≥65 years were identified from the Taiwan National Health Insurance Database. Patients receiving anti-arrhythmic drugs at a cumulative defined daily dose (cDDD) of >30 within the first year of enrollment constituted the rhythm control group. Patients who used rate control medications for a cDDD of >30 constituted the rate control group. A multivariate Cox hazards regression model was used to determine the hazard ratio (HR) for dementia. Results: A total of 3382 AF patients (698 in the rhythm control group; 2684 in the rate control group) were analyzed. During a 4.86 ± 3.38 year follow-up period, 414 dementia events occurred. The rhythm control group had a lower rate of dementia than the rate control group (adjust HR: 0.75, *p* = 0.031). The rhythm control strategy reduced the risk of dementia particularly in those receiving aspirin (*p* = 0.03). Conclusions: In patients with AF, pharmacological rhythm control was associated with a lower risk of dementia than rate control over a long-term follow-up period, particularly in patients receiving aspirin treatment.

## 1. Introduction

Atrial fibrillation (AF) and dementia both increase in prevalence with advancing age, and they share common risk factors, such as hypertension, diabetes mellitus, and coronary artery disease, in the elderly population [1]. AF is also a risk factor for cognitive impairment [2]. The pathological processes leading from AF to dementia are possibly multi-factorial, including cerebral hypo-perfusion, vascular inflammation, brain atrophy, genetic factors, and cerebral infarction with AF [3]. In addition to improving AF symptoms, restoring the sinus rhythm (the rhythm control strategy) is potentially a therapeutic option to lower the risk of dementia.

We and other groups have reported that the rhythm control of AF via catheter ablation (CA) lowered the risk of dementia in long-term follow-up cohorts, suggesting that rhythm control might prevent cognitive dysfunction [4,5]. Despite the number of AF patients receiving CA doubling over the last decade in Western countries, the availability of such therapy varies across regions, making pharmacological therapy the primary choice for rhythm control in AF patients [6]. Currently, pharmacological treatment remains the first-line therapeutic option for AF patients because no clear evidence is available to show the stroke/survival benefits with CA compared to medication in AF patients [7]. An AFFIRM study on AF patients reported that pharmacological rhythm control was not associated with a cognitive function improvement or survival benefit compared with rate control [8]. One possible explanation for the ineffectiveness of pharmacological rhythm control in reducing dementia risk in the AFFIRM trial is its relatively short (3.6 years) follow-up duration. Given that dementia is a slowly progressive disabling syndrome with cerebral degeneration, a longer duration follow-up is necessary to elucidate the protective effects of pharmacological treatment. Related to this issue, we and other groups have consistently found that pharmacological rhythm control reduced the risk of stroke in AF patients using long-term follow-ups [9,10]. Whether the reduced stroke risk with pharmacological rhythm control could translate into reduced dementia risk remains unclear. In this study, we hypothesized that pharmacological rhythm control for AF patients reduces the risk of dementia better than rate control. We then tested this hypothesis in a large population cohort with a long-duration follow-up.

## 2. Materials and Methods

### 2.1. Research Database

We used the National Health Insurance Research Database (NHIRD) of Taiwan for data collection. The database includes records of all outpatient visits, hospital admissions, prescriptions, and disease diagnoses [10]. We firstly obtained a subset of the NHIRD, which included one million subjects randomly selected from the period between 1999 and 2010. Since this research database included patients from 1999–2010, and was updated until 2013, the data of 2014–2020 were not included in this database. Subjects in this subset were representative of the general Taiwanese population. The International Classification of Diseases, Ninth Revision, Clinical Modification (ICD-9-CM) coding system has been used in the database since 2000 [11]. Our study was approved by the Institutional Review Board of Taichung Veterans General Hospital (CE13152B-4) in accordance with the Declaration of Helsinki and relevant guidelines/regulations. Since patient information is anonymous, the requirement for written informed consent was officially waived.

### 2.2. Study Population

Patients aged ≥65 years with a diagnosis of atrial flutter/fibrillation (AF) were identified (ICD-9-CM codes of 427.3, 427.31, and 427.32). Newly diagnosed AF was defined as three or more outpatient visits with a diagnostic code of AF within a year, or at least one hospitalization with a diagnostic code of AF [10]. The primary endpoints of this study were dementia, including senile/pre-senile dementia (ICD-9-CM 290.0–290.3, 294.1–294.2), arteriosclerotic dementia (ICD-9-CM 290.4), and Alzheimer’s disease (ICD-9-CM 331.0), determined according to the Diagnostic and Statistical Manual of Mental Disorders, 5th edition; DSM-V [4]. Patients with the above diagnoses at enrollment or those who died within the first year of enrollment were excluded.

Cardiovascular co-morbidities studied were hypertension, diabetes, heart failure, stroke, valvular heart disease (VHD), coronary artery disease (CAD), chronic obstructive pulmonary disease (COPD), and depression [11]. These were identified using the ICD-9-CM codes if the patient had at least one hospitalization or three consecutive outpatient visits under these diagnostic codes. The accuracy of diagnosis with these criteria has been validated in Taiwan’s NHIRD. For example, the positive predictive value for the diagnosis of AF was 89.0% [12]. The positive predictive values for co-morbidities such as heart failure, hypertension, and diabetes, were 97.6%, 88.5%, and 92.0%, respectively.

### 2.3. Definitions of Rhythm Versus Rate Control Strategies

Rhythm control in this study was mainly defined as the use of anti-arrhythmic drugs (AADs) to maintain sinus rhythm, although very few AF patients receiving cardioversion, AF ablation, or surgery were included [10]. Rate control was defined as use of any combination of β-blocker, calcium channel blocker, and digoxin without the intention for rhythm control. AF control strategies were determined at the discretion of the physician. Patients defined as the rhythm control group were those who had received any of the AADs for AF rhythm control and who had a cumulative defined daily dose (cDDD) of >30 (i.e., use a standard dose of any AAD for >30 days) within the first year of enrollment, regardless of the use of rate control medication. AADs and their classes for AF rhythm control included quinidine (Ia), propafenone (Ic), flecainide (Ic), amiodarone (III), and sotalol (III) [10]. Patients were defined as the rate control group if they received any of the rate control medications, such as beta-blockers, calcium channel blockers (diltiazem, verapamil), and digitalis for a cDDD of >30 within the first year of enrollment and had not received any AADs [10]. In this study, we used the cumulative defined daily dose (cDDD) as the medication dosage to improve the consistency of comparing different drugs. Therefore, patients who continuously, intermittently, or alternatively used rate/rhythm control medications for a cDDD of >30 within the first year of enrollment could fulfill the rate/rhythm control enrollment criteria. The current guideline recommended that the initial therapy for AF should always include rate control medications [7]. Even if the ultimate goal is rhythm control, rate control medications should be continued throughout follow-up. Therefore, patients receiving simultaneously both rhythm and rate control medications were classified as the rhythm control group. Information regarding medications such as prescribed drug types, dosages, dates of prescription, and total number of pills dispensed was obtained from an ambulatory and inpatient claims database.

### 2.4. Study Outcomes

The primary outcome of this study was dementia, including senile/pre-senile dementia, vascular dementia, and Alzheimer’s disease. The study endpoint was defined as any events which occurred after the patients being assigned to either group, including death, or the end of the follow-up date (31 December 2013) being reached.

### 2.5. Statistical Analysis

Continuous variables are presented as mean ± standard deviation, and categorical variables as proportions. For comparisons, an analysis of variance was used for continuous variables, and the Chi-square test was used for categorical variables. A propensity score was derived via logistic regression using age, gender, and comorbidities as independent variables. Multivariable Cox proportional hazard regression models were adjusted for age, gender, and comorbidities, with propensity scores for correcting confounding risk factors. A subgroup analysis was used to evaluate the dementia outcomes in AF patients with/without the specified co-morbidities and medications. The event-free survival curves were plotted using the Kaplan–Meier method, and results were compared using a log-rank test for statistical significance. The rate control group was designed as the reference, and the dementia risk in the rhythm control group was expressed by the hazard ratio (HR) and a 95% confidence interval (CI). All statistical analyses were performed with SAS software version 9.2 (SAS Institute, Inc., Cary, NC, USA). Statistically significant differences were set at *p* < 0.05.

## 3. Results

### 3.1. Baseline Characteristics

**Figure 1:**
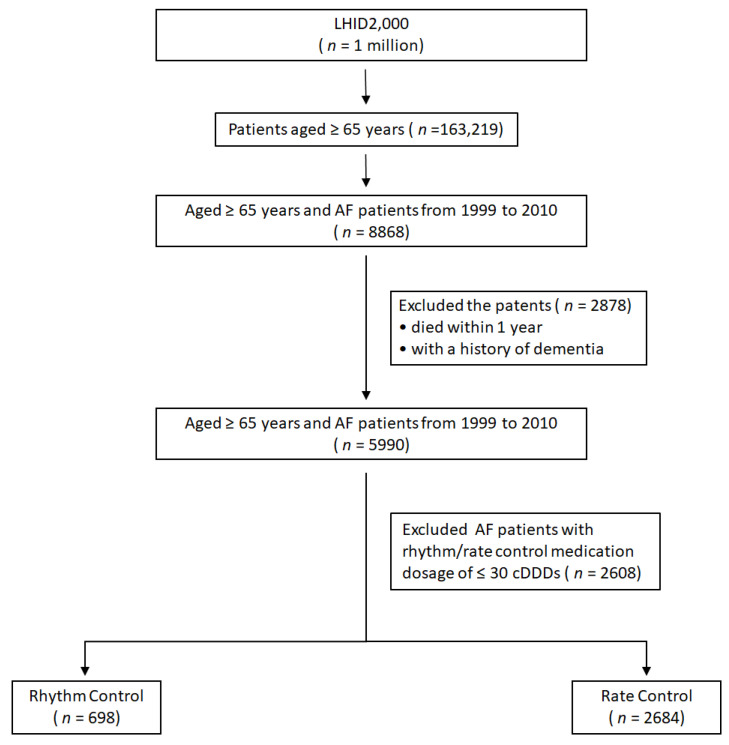
Flow chart of the study population. AF, atrial fibrillation; LHID, longitudinal health insurance database; cDDD, cumulative defined daily dose.

There were 163,219 patients aged ≥65 years in this cohort (Figure 1). A total of 8868 AF patients were identified within this group, with an AF prevalence of 5.43%. After applying the inclusion/exclusion criteria, 3382 of them were finally analyzed. There were 698 AF patients in the rhythm control group, and 2684 patients in rate control groups. Table 1 shows their baseline characteristics. Patients were older in the rate control group (*p* = 0.003). However, the percentage of patients aged ≥75 years (*p* = 0.09) and the gender distribution were similar across groups (*p* = 0.06). The percentages of patients with hypertension (*p* = 0.0008) and heart failure (<0.0001) were higher in the rate control group. However, percentages were similar across groups regarding their co-morbidities, such as diabetes mellitus (*p* = 0.36), stroke (*p* = 0.34), VHD (*p* = 0.56), CAD (*p* = 0.09), COPD (*p* = 0.58), and depression (*p* = 0.59). The propensity score in the rate control group (0.20 ± 0.05) was lower than that in the rhythm control group (0.22 ± 0.05) (*p* < 0.001).

More patients in the rate control group received angiotensin-converting enzyme inhibitors (ACEIs)/angiotensin receptor blockers (ARBs) (*p* = 0.0016), diuretics (*p* < 0.0001), aspirin (*p* = 0.03), and warfarin (*p* = 0.01). Fewer patients in the rate control group received clopidogrel (*p* = 0.0032) and statin (*p* = 0.0079). Aspirin was the most commonly used anti-thrombotic agent in both groups (~70%). The two groups had similar usages of alpha blockers (*p* = 0.30) and fibrate (*p* = 0.69). Similar percentages of patients received CA treatment for AF (*p* = 0.197), cardioversion (*p* = 0.165), and maze procedures (*p* = ns) in both groups. Table 2 lists the AADs used for the two groups. It shows that 1.4% of the rhythm control group was either not on rate control therapies or had a cDDD of ≤30 for rate control medication within the first year of enrollment.

### 3.2. AF Control Strategies and Factors on Dementia Risk

**Figure 2:**
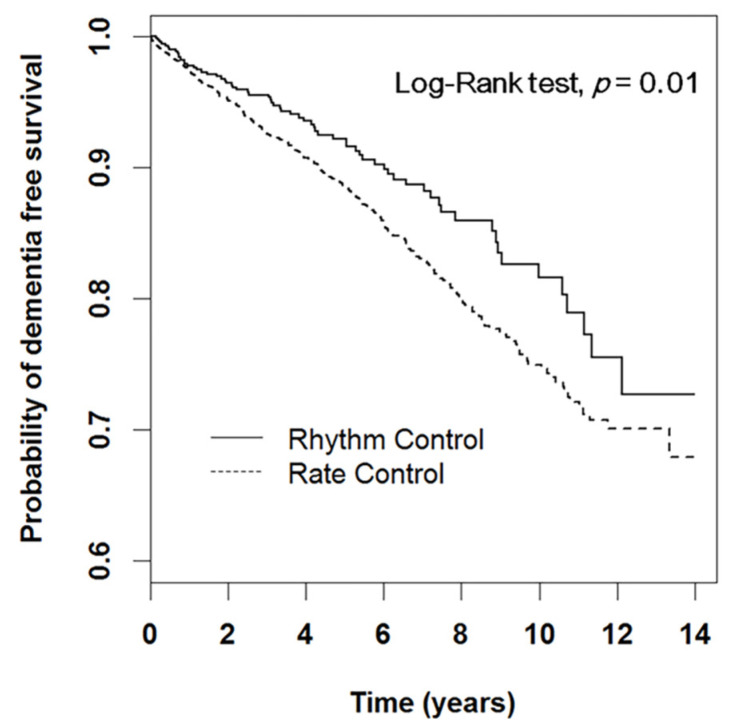
Kaplan–Meier survival curves for dementia outcomes in AF patients receiving rate and rhythm control strategies.

Within a mean follow-up duration of 4.86 ± 3.38 years, a total of 414 dementia events occurred. Table 3 shows the factors and HRs for dementia risk in both groups. Fewer dementia events occurred in the rhythm control group (19.1/1000 person-years (PYs)) than in the rate control group (26.8/1000 PYs) (adjusted HR 0.75, 95% CI 0.58–0.97, *p* = 0.031). Figure 2 shows the Kaplan–Meier survival curves for dementia risk between the two groups (log-rank *p* = 0.01).

In this cohort, a higher risk of dementia was associated with the rate control strategy (*p* = 0.031), ages ≥ 75 year (*p* = 0.002), and depression history (*p* = 0.012) than in those without these characteristics (Table 3). The rhythm control group (80.7/1000 PYs) had a lower mortality rate than that in the rate control group (113.7/1000 PYs) (*p* < 0.05).

### 3.3. Subgroup Strategy Analysis on Dementia Outcomes

Table 4 shows the subgroup analysis of AF control strategies regarding different patient characteristics for dementia risk. There was no significant difference in dementia risk between the two groups with the following: age (*p* = 0.42), sex (*p* = 0.18), hypertension (*p* = 0.54), diabetes (*p* = 0.11), heart failure (*p* = 0.19), stroke (*p* = 0.79), VHD (*p* = 0.66), and CAD (*p* = 0.07).

Other interactions were not significant: COPD (*p* = 0.76), depression (*p* = 0.26), use of clopidogrel (*p* = 0.35), ACEI/ARBs (*p* = 0.19), alpha blockers (*p* = 0.08), diuretics (*p* = 0.07), statins (*p* = 0.79), and fibrates (*p* = 0.83). In patients without anticoagulation (*n* = 590 and 2158 for the rhythm and rate control groups, respectively), the incidence rate of dementia was similar between the rhythm control (18.8/1000 PYs) and rate control (26.1/1000 PYs) groups (adjusted HR 0.79, 95% CI 0.59–1.06, *p* = ns). In patients with anticoagulation (*n* = 108 and 526 for the rhythm and rate control groups, respectively), the incidence rate of dementia was also similar between the rhythm control (20.9/1000 PYs) and rate control (29.9/1000 PYs) groups (adjusted HR 0.68, 95% CI 0.36–1.25, *p* = ns). The interaction between anticoagulation use and rhythm/rate control strategy was insignificant (*p* = 0.89).

In aspirin users, the rhythm control group showed a lower risk of dementia than the rate control group (adjusted HR 0.64). In non-aspirin users, no difference was found on dementia risk between the two groups.

### 3.4. Matched Cohort

The baseline characteristics of both groups were quite different. To reduce the confounding bias, we matched the two groups with age and gender on a 1:1 ratio to evaluate the study outcomes (Table 5). In this 1:1 matched cohort, the rhythm control group (*n* = 698) showed a lower risk of dementia than the rate control group (*n* = 698) (adjusted HR 0.66, 95% CI 0.48–0.90, *p* = 0.009).

## 4. Discussion

The major findings in this study are: (1) the rhythm control strategy in patients with AF was associated with a lower risk of dementia compared to patients receiving a rate control strategy; (2) aspirin use with the rhythm control strategy had a synergic effect to reduce the risk of dementia.

### 4.1. Risk Factors for Dementia in AF Patients

The relationship between AF and dementia has been reported, as AF increases the risk of stroke by four or five times. Prior stroke, cerebral hypoperfusion, micro-emboli, micro-bleeds, and systemic inflammation may also contribute to dementia being related to AF [13]. AF patients without prior stroke history also show higher risks of dementia [14]. The incidence of dementia increases with age, from 0.1% at age 60–64 to >8% at age 95, indicating that age is a strong risk factor of dementia [15]. We also confirmed that our patients aged ≥75 had a higher dementia risk than those aged <75 years (Table 3).

Wändell et al. reported that depression is an independent risk factor for dementia, particularly in male AF patients, although survival bias could have existed in that study [16]. We also found that depression is a risk factor for dementia (adjusted HR 2.22) in our cohort, in which >50% of patients in both groups were male. On the other hand, Bellomo et al. found that AF patients have higher risks of both cognitive dysfunction and depression, regardless of stroke [17]. The causative relationship among AF, depression, and dementia remains to be investigated.

### 4.2. Rhythm Versus Rate Control on Dementia Risk

AF has been reported to be associated with decreased cerebral blood flow and brain perfusion [18]. Gardarsdottir et al. in a large cohort, found that cerebral blood flow and brain perfusion in patients with persistent AF were lower than those in patients with sinus rhythms [18]. This notion was further supported by a study finding that the restoration and maintenance of sinus rhythm after elective cardioversion for AF was associated with improved brain perfusion and cerebral blood flow [19]. By using a non-invasive spatially resolved cerebral near-infrared spectroscope, Saglietto et al. found that sinus rhythm restoration via electrical cardioversion in AF patients significantly reduced the burden of extreme single-beat hemodynamic events in cerebral microcirculation [20]. These findings indicated that restoring sinus rhythm (rhythm control) might reduce the beat-to-beat variance in cerebral blood flow, prevent cerebral hypo-perfusion and silent cerebral infarction, and lead to the prevention of dementia [18,19,20]. This speculation is supported by the findings that rhythm control by CA lowers the risks of dementia in our and others’ AF cohorts with long-term follow-ups [4,5]. However, regional differences in the availability of CA exist, making pharmacological therapy the primary choice for rhythm control in AF patients [6]. Furthermore, according to the current AF guidelines, medical therapy remains the first-line therapeutic option [7].

In the cornerstone AFFIRM study on AF patients, pharmacological rhythm control showed no benefits on cognitive function or survival over rate control, with the study based on a 3.6-year follow-up [8]. Since dementia is a progressive disabling syndrome, a longer duration might be necessary to elucidate the protective effects of rhythm control on dementia in AF patients. A previous study showed that ~85% of dementia occurred at the age of 75 years and older [21]. An observational study also found that mild cognitive impairment might progress to dementia within 5 years [21]. In this study, the average ages in both groups were ~75 years, an age with a high prevalence of dementia. The follow-up duration in this study was 4.86 ± 3.38 years, which might allow dementia to develop within 5 years, and should be long enough to find the difference in dementia between two groups.

In our cohort, <2% of AF patients had received CA for AF, cardioversion, or a maze procedure, indicating that this is a representative pharmacological rhythm control cohort. We observed that pharmacological rhythm control was associated with a lower risk of dementia. Even though the higher mortality rate in the rate control group might prevent the detection of dementia and under-estimate the actual dementia rate, the rhythm control group still showed an even lower risk of dementia, suggesting a robust effect of rhythm control in reducing dementia risk. Our present study, together with previous studies reporting similar results, pointed to the benefits of rhythm control, either using CA or medication, which could reduce the risk of dementia [4,5].

### 4.3. Aspirin Use on Dementia Risk in Patients with AF

The underlying mechanisms linking AF to cognitive dysfunction could involve systemic inflammation, cerebral small vessel diseases, hypoperfusion, and micro-emboli [13]. The anti-inflammatory and anti-thrombotic properties of aspirin raise the speculation that aspirin use might prevent cognitive dysfunction in AF patients. However, compared with other anticoagulants, aspirin is ineffective in the prevention of stroke, which was a major cause of vascular dementia. Aspirin also carries a potential harm of bleeding events that might lead to dementia in AF patients. A recent large meta-analysis on the elderly population without AF also reported that the chronic use of low-dose aspirin did not improve cognitive function [22].

In this study, AF patients in the rhythm control group showed lower risks of dementia after adjusting the baseline characteristics. However, the subgroup analysis showed that the rhythm control strategy reduced the risk of dementia, particularly in AF patients who were aspirin users. In addition, a higher percentage of aspirin users were found in the rhythm control group (72.8%) than in the rate control group (68.6%) (*p* = 0.03). The mechanisms by which aspirin might potentiate the benefit of rhythm control in reducing dementia risks remain unclear. AF is associated with systemic inflammation and increased circulating inflammatory mediators, including interleukin-6 and tumor necrosis factor-α [23]. Recurrent episodes of AF might lead to inflammatory structural/electrical remodeling within the atria and the perpetuation AF, known as the “AF begets AF” hypothesis. In a mouse model of Alzheimer’s disease, aspirin could inhibit inflammatory gene PPARα expression and attenuate amyloid plaque pathology in brain cells. It is possible that the reduction of the AF burden with rhythm control together with anti-inflammatory aspirin use might mutually contribute to inhibit more systemic inflammatory responses and decrease the dementia risk more so than rate control, allowing AF to recur. Further prospective studies are needed to elucidate the role of aspirin on such dementia prevention in AF patients.

## 5. Limitations

Firstly, this is a retrospective observational cohort study, and the diagnosis of dementia was obtained according to the ICD-9-CM codes registered by physicians and re-confirmed by a certified coding specialist. We could not completely exclude the possibility of miscoding. Since patients’ information is anonymous, detailed information regarding the National Institute on Aging and Alzheimer’s Association (NIA-AA) scores for the diagnosis of dementia along with the image studies were not available [24]. Secondly, the subtypes of AF (paroxysmal, persistent, and permanent) and AF duration could not be differentiated using the ICD-9-CM codes, and this was a limitation as in our previous studies [10,25]. Whether AF subtypes might affect pharmacological rhythm control efficacy remains unclear. Thirdly, the cohort was quite outdated, as demonstrated by the high aspirin use rate before non-vitamin K oral anticoagulant drug (NOAC) was available for stroke prevention. Whether our results can be applied to the era of NOAC remains to be explored. Fourthly, the patient numbers and baseline characteristics (e.g., age, hypertension, heart failure, and medications used) were not evenly distributed in both groups, indicating that heterogeneities might exist between both groups. The reason why the age of patients in the rate control group was higher than that in the rhythm control group remained unclear. One possible explanation could be that the physicians tended to adapt the rate control strategy to older patients because rate control medications were associated with less adverse effects compared to rhythm control medications. To reduce the confounding bias, we built another cohort by matching the two groups with age and gender in a 1:1 ratio. In this matched cohort, the rhythm control group consistently showed a lower risk of dementia than rate control (*p* = 0.009). This finding might support the main conclusion in the original study cohort. Finally, despite having adjusted the multivariable Cox hazards regression model according to baseline characteristics, co-morbidities, and propensity scores, along with performing a subgroup analysis to minimize the impact of selection bias on study outcomes, our results should be interpreted with caution because the baseline medications used were not adjusted in the regression model, and the risk factors in the matched cohort were not completely eliminated. In this study, rhythm control was defined as any use of AADs on top of rate control therapy as mandated by guidelines. Therefore, we cannot discount any additive/synergistic effects of the combination of therapies resulting in better outcomes in rhythm over the rate control cohort.

## 6. Conclusions

The pharmacological rhythm control strategy was associated with a lower risk of dementia in elderly (aged >65 years) AF patients compared to the rate control strategy following a long-term follow-up. The use of aspirin might reduce the dementia risk in AF patients undergoing pharmacological rhythm control. Further research is needed to elucidate the effect of rhythm versus rate control on dementia risk in AF patients receiving NOACs.

## Figures and Tables

**Table 1:** Baseline characteristics of the AF patients.

Variables	Rhythm Control	Rate Control	*p*-Value
	*N* = 698	*N* = 2684	
Age, mean (SD)	75.1 (6.2)	75.9 (6.7)	0.003 *
≥75 (%)	362 (51.9)	1489 (55.5)	0.09
Male (%)	398 (57.0)	1425 (53.1)	0.06
Co-morbidity (%)			
Hypertension	563 (80.7)	2303 (85.8)	0.0008
Diabetes	216 (30.9)	879 (32.7)	0.36
Heart failure	214 (30.7)	1186 (44.2)	<0.0001
Stroke	271 (38.8)	1095 (40.8)	0.34
VHD	165 (23.6)	663 (24.7)	0.56
CAD	478 (68.5)	1925 (71.7)	0.09
COPD	403 (57.7)	1581 (58.9)	0.58
Depression	49 (7.0)	173 (6.4)	0.59
** Propensity score	0.22 (0.05)	0.20 (0.05)	<0.001
Medications (%)			
ACEIs/ARBs	427 (61.2)	1812 (67.5)	0.0016
Alpha blockers	147 (21.1)	518 (19.3)	0.3
Diuretics	379 (54.3)	1831 (68.2)	<0.0001
Aspirin	479 (68.6)	1953 (72.8)	0.03
Clopidogrel	116 (16.6)	332 (12.4)	0.0032
Warfarin	108 (15.5)	526 (19.6)	0.01
Statins	131 (18.8)	394 (14.7)	0.0079
Fibrates	28 (4.01)	117 (4.36)	0.69
Catheter ablation for AF (%)	7 (0.26)	4 (0.57)	0.197
Electrical cardioversion (%)	9 (1.3)	20 (0.7)	0.165
Maze procedure (%)	0 (0%)	1 (0%)	-

* Determined with Student’s t test. ** Propensity score was derived via logistic regression using age, gender, and co-morbidities as independent variables. AF, atrial fibrillation; CAD, coronary artery disease; COPD, chronic obstructive pulmonary disease; VHD, valvular heart disease; ACEI, angiotensin-converting enzyme inhibitor; ARB, angiotensin receptor blocker.

**Table 2:** Medications used for rate and rhythm control in AF patients.

Medications	Rhythm Control *N* = 698	Rate Control *N* = 2684	*p*-Value
Rate control (%)			
β-Blocker	299 (42.8)	1700 (63.3)	<0.0001
Diltiazem	145 (20.8)	1060 (39.5)	<0.0001
Verapamil	62 (8.9)	405 (15.1)	<0.0001
Digoxin	182 (26.1)	1860 (69.3)	<0.0001
Rhythm control (%)			
Quinidine	4 (0.57)		
Flecainide	4 (0.57)		
Propafenone	255 (36.5)		
Amiodarone	540 (77.4)		
Sotalol	6 (0.86)		

**Table 3:** Factors and the hazard ratio for dementia in AF patients.

Variable	Event	PYs	Event Rate	Crude HR (95% CI)	Adjusted HR (95% CI)	*p*-Value
AF control strategy						
Rate control	347	12,936	26.8	ref	ref	
Rhythm control	67	3501	19.1	0.71 (0.55–0.92)	0.75 (0.58–0.97)	0.031
Age						
<75	155	9024	17.2	ref	ref	
≥75	259	7412	34.9	2.14 (1.75–2.62)	1.72 (1.23–2.42)	0.002
Sex						
Female	227	7459	30.4	ref	ref	
Male	187	8977	20.8	0.68 (0.56–0.83)	0.91 (0.58–1.42)	0.667
Hypertension						
No	53	2744	19.3	ref	ref	
Yes	361	13,693	26.4	1.38 (1.03–1.84)	0.51 (0.13–2.03)	0.339
Diabetes						
No	280	11,423	24.5	ref	ref	
Yes	134	5014	26.7	1.10 (0.90–1.36)	0.96 (0.78–1.19)	0.706
Heart failure						
No	251	10,423	24.1	ref	ref	
Yes	163	6014	27.1	1.15 (0.94–1.40)	0.29 (0.03–2.5)	0.262
Stroke						
No	218	10,446	20.9	ref	ref	
Yes	196	5990	32.7	1.59 (1.31–1.93)	1.25 (0.95–1.66)	0.116
VHD						
No	315	12,694	24.8	ref	ref	
Yes	99	3742	26.5	1.07 (0.86–1.35)	1.23 (0.79–1.91)	0.354
CAD						
No	103	4742	21.7	ref	ref	
Yes	311	11,695	26.6	1.22 (0.98–1.53)	0.99 (0.76–1.28)	0.930
COPD						
No	159	7059	22.5	ref	ref	
Yes	255	9378	27.2	1.21 (1.00–1.48)	1.2 (0.94–1.54)	0.142
Depression						
No	372	15,473	24.0	ref	ref	
Yes	42	963	43.6	1.84 (1.34–2.53)	2.22 (1.19–4.13)	0.012

Model adjusted for age, sex, co-morbidities, and propensity score. Rate is expressed as per 1000 person-years (PYs).

**Table 4:** Subgroup analysis of the hazard ratio for dementia in AF patients.

	Rate Control		Rhythm Control		Adjusted HR (95% CI)	*p* for Interaction
Variable	Event	Rate	Event	Rate		
Age						0.42
<75	131	18.7	24	12.0	0.65 (0.42–1.01)	
≥75	216	36.5	43	28.7	0.79 (0.57–1.10)	
Sex						0.18
Female	197	33.0	30	20.0	0.63 (0.43–0.93)	
Male	150	21.5	37	18.5	0.93 (0.65–1.34)	
Hypertension						0.54
No	40	20.0	13	17.5	0.91 (0.48–1.74)	
Yes	307	28.1	54	19.6	0.74 (0.55–0.99)	
Diabetes						0.11
No	238	26.8	42	16.5	0.65 (0.47–0.90)	
Yes	109	26.9	25	26.1	1.05 (0.68–1.63)	
Heart failure						0.19
No	199	25.3	52	20.3	0.90 (0.66–1.22)	
Yes	148	29.2	15	15.9	0.54 (0.31–0.91)	
Stroke						0.79
No	183	22.3	35	15.5	0.76 (0.53–1.10)	
Yes	164	34.6	32	25.7	0.80 (0.55–1.17)	
VHD						0.66
No	265	26.6	50	18.4	0.73 (0.54–0.99)	
Yes	82	27.7	17	21.8	0.94 (0.55–1.60)	
CAD						0.07
No	78	21.6	25	22.3	1.12 (0.71–1.78)	
Yes	269	28.9	42	17.7	0.65 (0.47–0.90)	
COPD						0.76
No	133	24.2	26	16.6	0.70 (0.46–1.07)	
Yes	214	28.7	41	21.2	0.81 (0.58–1.14)	
Depression						0.26
No	314	25.8	58	17.6	0.73 (0.55–0.97)	
Yes	33	43.2	9	45.2	1.27 (0.59–2.77)	
Aspirin						0.03
No	76	20.5	25	22.9	1.19 (0.75–1.87)	
Yes	271	29.4	42	17.5	0.64 (0.46–0.89)	
Clopidogrel						0.35
No	318	27.3	62	20.5	0.80 (0.61–1.05)	
Yes	29	22.7	5	10.6	0.63 (0.24–1.67)	
Warfarin						0.89
No	274	26.1	55	18.8	0.79 (0.59–1.06)	
Yes	73	29.9	12	20.9	0.68 (0.36–1.25)	
ACEIs/ARBs						0.19
No	101	21.6	27	19.5	0.99 (0.64–1.52)	
Yes	246	29.8	40	18.9	0.67 (0.48–0.94)	
Alpha blockers						0.08
No	272	25.9	57	20.7	0.86 (0.64–1.14)	
Yes	75	30.9	10	13.4	0.48 (0.25–0.93)	
Diuretics						0.07
No	103	21.2	37	20.4	1.14 (0.78–1.68)	
Yes	244	30.3	30	17.7	0.56 (0.39–0.83)	
Statins						0.79
No	310	28.0	58	19.9	0.76 (0.58–1.01)	
Yes	37	19.7	9	15.3	0.74 (0.35–1.58)	
Fibrates						0.83
No	334	27.1	65	19.2	0.76 (0.58–0.99)	
Yes	13	20.9	2	17.4	1.03 (0.2–5.3)	

Model adjusted for age, sex, hypertension, diabetes, heart failure, stroke, VHD, CAD, COPD, and depression. PYs: person-years. Event rate is expressed as per 1000 PYs.

**Table 5:** Baseline characteristics of the matched cohort.

Variables	Rhythm Control *N* = 698 (%)	Rate Control *N* = 698 (%)	*p*-Value
Age, mean (SD)	75.1 (6.2)	75.1 (6.3)	0.983
≥75	362 (51.9)	362 (51.9)	1.000
Male	398 (57.0)	398 (57.0)	1.000
Co-morbidity			
Hypertension	563 (80.7)	595 (85.2)	0.023
Diabetes	216 (30.9)	227 (32.5)	0.527
Heart failure	214 (30.7)	289 (41.4)	<0.001
Stroke	271 (38.8)	282 (40.4)	0.547
VHD	165 (23.6)	176 (25.2)	0.493
CAD	478 (68.5)	512 (73.4)	0.045
COPD	403 (57.7)	408 (58.5)	0.786
Depression	49 (7.0)	36 (5.2)	0.146

This cohort was matched with age and gender on a 1:1 ratio from the original cohort. VHD, valvular heart disease; CAD, coronary artery disease; COPD, chronic obstructive pulmonary disease.

## Data Availability

All relevant data are within the paper.
